# Assessing Genomic Competencies: Insights From the Genetics and Genomics Nursing Practice Survey (GGNPS)

**DOI:** 10.1155/nrp/6680050

**Published:** 2026-07-22

**Authors:** Mahmoud Issam Salameh, Amani Anwar Khalil

**Affiliations:** ^1^ Emirates Health Services, Alqassimi Hospital, Sharjah, UAE; ^2^ School of Nursing, The University of Jordan, Amman, Jordan, ju.edu.jo; ^3^ College of Nursing, RAK Medical and Health Sciences University, Ras Al-Khaimah, UAE

**Keywords:** attitude, competency, confidence, decision, genetics, genomics, knowledge, nursing education, nursing practice

## Abstract

**Introduction:**

In ICU settings, genomic information can contribute to diagnostic clarification, individualized treatment planning, medication optimization, and appropriate referral to specialized genetic services. Critically ill patients frequently present with complex multisystem conditions, inherited disorders, or unexplained clinical manifestations that may have underlying genetic components. Consequently, genomic literacy among ICU nurses is particularly important to support family history assessment, recognition of genetic risk factors, patient education, and genomics‐informed clinical decision‐making. However, translating genomic knowledge into effective nursing practice requires not only foundational genomic knowledge but also positive attitudes, adequate confidence, and strong clinical decision‐making skills among nurses.

**Purpose:**

This study evaluates the knowledge, attitudes, receptivity, confidence, and practices related to genetics and genomics among registered nurses working in ICUs in Amman, Jordan.

**Methods:**

A cross‐sectional, descriptive study was conducted with 229 ICU nurses recruited conveniently from three hospitals in Amman. Participants completed the Genetics and Genomics in Nursing Practice Survey (GGNPS).

**Results:**

The Jordanian nurses demonstrated adequate knowledge/competencies of genetics and genomics concepts, with an average score of 66.9% on the competency subscale. They had a positive attitude toward integrating genetics into their practice and exhibited high confidence in accessing and sharing genetic information. Clinical application remained limited, with 82.1% of providers rarely or never collecting a comprehensive family history. Adoption of genetics and genomics in practice was influenced by providers’ intent to expand their knowledge and their perceptions of the value and relevance of family history in patient care. Most nurses expressed a desire to learn more about genetics, especially if supported by senior nurses. A significant positive correlation was found between knowledge and confidence scores (*r* = 0.197, *p* = 0.003), indicating that nurses with higher levels of genomic knowledge reported greater confidence in their ability to discuss common genetic diseases and family history.

**Conclusion:**

While nurses possessed adequate knowledge and positive attitudes, their practical application of genetic information, particularly in family history assessment, was limited. A strong desire for further, peer‐supported education was observed. These findings emphasize the need for targeted training programs to bridge the gap between knowledge and practice, ultimately enhancing the quality of genetic‐informed nursing care in the ICU.

## 1. Introduction

Genetics and genomics‐related conditions can directly or indirectly lead to high rates of morbidity and mortality [1]. Genetic and genomic technologies are increasingly being integrated into routine patient care across various healthcare settings to improve outcomes [2, 3]. This includes areas such as pharmacogenetics, genetic cancer risk assessment, and newborn screening [2, 4]. The integration of genetics and genomics into intensive care practice is becoming increasingly important with the advancement of precision medicine, rapid genomic testing, pharmacogenomics, and genetic risk stratification. While genomic technologies may directly improve diagnosis, screening, and treatment selection, nurses’ genomic competencies primarily support implementation processes, including family history collection, genomic risk recognition, patient education, communication, and referral facilitation. Jordan has one of the highest rates of endogamy among Arab countries, which is associated with a higher incidence of both recognized and novel genetic disorders [5, 6]. Clinical genetic services enhance patient and family outcomes by diagnosing rare or recurrent genetic diseases using family history and medical records. Despite the prevalence of genetic diseases in Jordan due to high endogamy rates, genetic services in the country remain limited [1]. Still, courses in genetic science are not included sufficiently in the theory and clinical practice of the nursing curriculum [7, 8].

The prevention and management of common complex diseases, such as cardiovascular diseases and diabetes, which have well‐established genetic components, are considered a challenge to healthcare providers in various settings. Integrating genomic risk assessment into routine care can help identify at‐risk individuals and tailor prevention strategies [9–11]. Genetic research has enhanced our comprehension of numerous disorders that result in critical illness, including cardiomyopathies, certain malignancies, and bad drug reactions (pharmacogenomics). In the ICU, where patients are exceptionally vulnerable, prompt and precise information is crucial. A patient’s genetic profile can affect their response to drugs such as warfarin or clopidogrel, hence influencing therapy efficacy and safety. Additionally, a familial history of illnesses such as long QT syndrome or inherited cardiomyopathies, which may result in abrupt cardiac incidents, is essential for thorough patient evaluation. Consequently, ICU nurses, positioned at the front of patient evaluation and surveillance, necessitate essential genetic competencies to identify at‐risk individuals, guide clinical decision‐making, and deliver safe, individualized treatment.

The genetic services available in Jordan are limited and not tailored to the population’s specific needs [7]. Nurses play a pivotal role in meeting the growing demand for genetic and genomic healthcare services [1]. The present study assessed the genetic knowledge and practices of Jordanian ICU nurses, highlighting the need to enhance their education, practice, and healthcare services to reduce morbidity and mortality. The Genetics and Genomics Nursing Practice Survey (GGNPS) is a validated instrument used globally to assess nurses’ knowledge, attitudes, and integration of genetics and genomics into their practice. This broad applicability holds significant value for advancing nursing education and practice worldwide [10].

## 2. Aims of the Study

1. To assess ICU nurses’ genetics and genomics knowledge, attitudes/receptivity, decision/adoption, and confidence regarding genomics‐informed nursing practice, including their ability to identify relevant family history information for assessing patients’ genetic susceptibility to common diseases.
2. To examine the associations between ICU nurses’ genetics and genomics knowledge and their demographic and work‐related characteristics, attitudes/receptivity, decision/adoption, and confidence in supporting genomics‐informed nursing care, including recognition of genetic risk and referral to appropriate genetic counseling or genomic services when indicated.

## 3. Methodology

### 3.1. Design

This is a cross‐sectional descriptive study conducted among registered nurses during their duty at the ICU. The nurses filled out the GGNPS.

### 3.2. Sample and Sampling

A total population (census) sampling approach was used, whereby all eligible ICU nurses were invited to participate from September until December 2020. Recruitment was conducted via institutional communication channels, and participation was voluntary. Inclusion criteria included full‐time registered nurses with a BSc degree providing direct patient care in medical or surgical ICUs. Full‐time nurses were incorporated to guarantee uniformity in clinical exposure, workload, and proficiency with ICU protocols and patient care procedures. Full‐time personnel are more inclined to maintain ongoing involvement with critically sick patients, which is vital for appropriately evaluating the factors under investigation.

### 3.3. Settings

Three hospitals were purposefully selected to represent the three main healthcare sectors in Jordan: a large government‐funded hospital, a university‐affiliated (educational) hospital, and a private hospital. These specific hospitals were chosen because they have the largest ICU capacities in Amman, ensuring access to a large and diverse sample of ICU nurses. Amman was chosen as the setting because it is the capital and largest city, housing approximately 42% of Jordan’s population. This makes its hospital network a major provider of tertiary and critical care services for the country and its nursing workforce potentially representative of the national context.

### 3.4. Measures

The GGNPS was originally developed and published by Calzone et al. to assess registered nurses’ knowledge, attitudes, and integration of genetics and genomics into practice [12]. The instrument has undergone rigorous psychometric testing, including test–retest reliability and content validity established by an expert panel, construct validity analysis, and test–retest reliability [12–14]. Reliability testing revealed that shorter Likert scales were more effective, with Cronbach’s alpha coefficients ranging from 0.71 to 0.89 across subscales in previous studies [14].

The GGNPS consists of six subscales: (1) Demographic and Clinical Profile; (2) Attitudes and Receptivity; (3) Confidence; (4) Competency/Knowledge; (5) Social System; and (6) Decision and Adoption [12]. The final instrument contains 43 questions comprising multiple‐choice, dichotomous *yes*/*no*, and Likert scale items.

For the current study, the survey was administered in English, as English is the primary language of nursing education and documentation in Jordanian healthcare settings. All participating nurses were proficient in English, having completed their nursing education in English‐medium programs. Therefore, no translation was required, and the original psychometric properties were maintained. However, we conducted internal consistency testing for the current sample, which yielded acceptable reliability (Cronbach’s *α* = 0.76 for the knowledge subscale).

### 3.5. Data Collection Procedure

Subsequent to obtaining ethical approval, the principal investigator reached out to the nursing directors of the chosen institutions to elucidate the study’s objectives and secure institutional consent. Subsequently, the researcher visited the ICUs during staff shift changes and briefings to elucidate the study to prospective volunteers. Nurses were selected via convenience sampling.

Data were gathered by a self‐administered, paper‐based questionnaire. The GGNPS was utilized in its original English form, as English serves as the principal language of education and documentation in all Jordanian nursing programs and healthcare environments. Consequently, translation was unnecessary.

The researcher supplied each participant with an information sheet outlining the study’s objectives, methodologies, risks, and advantages, underscoring that participation was voluntary and confidential. Informed consent was obtained in writing from all participants prior to their completion of the questionnaire. The researcher was present on‐site to address any inquiries. Participants submitted completed questionnaires into a sealed box to maintain confidentiality.

The study employed a convenience sampling approach, including all eligible ICU nurses from the three selected institutions. A post hoc power analysis was conducted using G∗Power to evaluate the adequacy of the sample size. With a total sample of 229 participants, the study achieved a statistical power of 0.85 to detect a small‐to‐moderate effect size (*r* = 0.20) at a significance level of *α* = 0.05. This indicates that the sample size was sufficient to support the planned correlation analyses.

### 3.6. Ethical Consideration

The study received ethical approval from the University of Jordan (Reference No. 10‐2020‐20536) and participating hospitals. Informed consent was obtained, and confidentiality/anonymity was ensured through data coding and private questionnaire completion.

### 3.7. Data Analysis Plan

The Statistical Package for the Social Sciences (SPSS) Version 21 was used for data analysis. Data entry was double‐checked to prevent errors, and missing data and outliers were cleaned using frequency checks and central tendency measures. Descriptive statistics, including mean and standard deviation, were calculated.

Total scores for the main variables (knowledge and competency) were computed, and normality was assessed using Fisher’s skewness and histograms for continuous variables (knowledge scores, age, nursing experience, and ICU experience). Differences in knowledge and competency across gender, educational levels, work areas, and decision/adoption questions were analyzed using independent *t*‐tests, as this test is suitable for comparing means of normally distributed continuous variables between two independent groups. Relationships between knowledge/competency, age, experience, and confidence total score were examined using Pearson correlation tests, as it is appropriate for assessing linear associations between normally distributed continuous variables.

## 4. Results

### 4.1. The Sample Characteristics

A total of 460 surveys were distributed. We received 250 questionnaires (a 54.3% return rate). Of those, 21 were excluded due to significant missing data (more than 10% of items incomplete). Therefore, 229 fully completed surveys were included in the final analysis. The 49.7% represents the final analyzable response rate (229/460).

Only 229 out of 460 were returned with no missing data, with a total response rate of 49.7%. Nurses were recruited from different hospitals: 110 (48%) from the governmental, 90 (39.3%) from an educational, and 29 (12.7%) from the private hospital (Table 1). One hundred and seventeen nurses (51.1%) were females. The mean age was 31.2 (SD = 3.64) (range 24–42). Most nurses (*n* = 195, 85.2%) had baccalaureate degrees, and 34 nurses (14.8%) held a master’s degree. The mean percent of work time spent taking care of the patients is 75.79% (SD = 1.24) (range 30%–95%); the mean total number of years of nursing experience is 7.93 (SD = 3.42) (range 2–20).

**TABLE 1:** Demographic and work‐related characteristics of participants (*N* = 229).

Characteristic	*n* (%) or mean ± SD
Age, years	31.21 ± 3.65
Age range	24–42
Gender	
Male	112 (48.9%)
Female	117 (51.1%)
Highest nursing degree	
Baccalaureate degree in nursing	195 (85.2%)
Master’s degree in nursing	34 (14.8%)
Hospital types	
Governmental	110 (48%)
Educational	90 (39.3%)
Private	29 (12.7%)
Years worked in nursing	7.93 ± 3.42
Years worked in nursing, range	2–20
Current primary functional area	
Patient care	229 (100.0%)
Current primary area of practice/expertise	
Staff nurse	229 (100.0%)
Work time spent taking care of patients	75.79% ± 12.44%
Work time spent taking care of patients, ranges	30%–95%

### 4.2. Competency/Knowledge Subscale

The Competency/Knowledge scores demonstrated an approximately normal distribution based on visual inspection of histograms and Q–Q plots. The mean knowledge score was 8.03 out of 12 (SD = 1.995), corresponding to 66.9%, indicating a moderate level of genomic competency among ICU nurses.

Analysis of individual items revealed a distinct pattern of strengths and knowledge gaps (Table 2). Nurses showed strong recognition of the clinical importance of genetics, with the highest correct response rate observed for the item *Family history taking should be a key component of nursing care* (94.3%). Additionally, a high proportion of participants correctly identified the relevance of genetic risk in common diseases such as diabetes, cardiovascular disease, and cancer, suggesting adequate conceptual awareness of genomics in disease etiology.

**TABLE 2:** Knowledge/competencies of genetics and genomics among critical care nurses score (*N* = 229).

Item number	Item	Correct, *n* (%)	Incorrect, *n* (%)
1	A family history that includes only 1st‐degree relatives such as parents, siblings, and children should be taken on every new patient.	58 (25.3)	171 (74.7)
2	A family history that includes 2nd and 3rd‐degree relatives such as grandparents, aunts, uncles, and cousins should be taken for every new patient.	188 (82.1)	41 (17.9)
3	Family history taking should be a key component of nursing care.	216 (94.3)	13 (5.7)
4	There is a role for nurses in counseling patients about genetic risks.	198 (86.5)	31 (13.5)
5	Do you think that genetic risk (e.g., as indicated by family history) has clinical relevance for breast cancer?	168 (73.4)	6 (26.6)
6	Do you think that genetic risk (e.g., as indicated by family history) has clinical relevance for colon cancer?	164 (71.6)	65 (28.4)
7	Do you think that genetic risk (e.g., as indicated by family history) has clinical relevance for coronary heart disease?	170 (74.2)	59 (25.8)
8	Do you think that genetic risk (e.g., as indicated by family history) has clinical relevance for diabetes?	187 (81.7)	42 (18.3)
9	Do you think that genetic risk (e.g., as indicated by family history) has clinical relevance for ovarian cancer?	182 (79.5)	47 (20.5)
10	Extent to which family history supports clinical decisions (such as administering drugs prescribed).	186 (81.2)	43 (18.8)
11	The DNA sequences of two randomly selected healthy individuals of the same sex are 90%–95% identical.	54 (23.6)	175 (76.4)
12	Most common diseases such as diabetes and heart disease are caused by a single gene variant.	69 (30.1)	160 (69.9)

In contrast, foundational genomic knowledge and application‐specific understanding were notably weaker. The lowest performance was observed in items assessing core genetic principles, particularly the statement regarding DNA similarity between individuals (23.6% correct), as well as misconceptions related to the genetic basis of common diseases and limitations of family history restricted to first‐degree relatives (Table 2). These findings indicate gaps in fundamental genomic literacy, rather than deficiencies in general awareness.

Importantly, the results highlight a disconnect between recognition of importance and depth of understanding. While nurses largely appreciate the role of genetics in patient care, their ability to accurately interpret and apply genomic concepts remains limited. This pattern suggests that current knowledge is surface‐level and insufficient for clinical integration, particularly in critical care settings where nuanced interpretation of genetic information may influence decision‐making.

Overall, the competency findings suggest that although ICU nurses possess baseline awareness of genomic relevance, there is a clear need for targeted educational interventions focusing on core genetic concepts and their clinical application, rather than solely increasing general exposure to the topic.

### 4.3. Attitudes of Genetics

The Attitudes and Receptivity domain covers the perceived importance, advantages, and disadvantages of integrating genomics into practice; the complexity of integrating family history into practice; and the perception of the value of family history in patient care and personal practice [12, 13].

Most respondents felt it was essential (70.7%; *n* = 162/229) or somewhat important (15.7%; *n* = 36/229) to become more educated about the genomics of common diseases. The highest‐rated positive attitude was agreement that integrating the genetics of common diseases into clinical practice enhances treatment decision‐making (80.3%, *n* = 184/229). The lowest advantage of integrating genetics of common diseases into practice is to improve services to the patients, with a percentage of 64.6% (Table 3).

**TABLE 3:** Attitudes toward genetics and genomics among critical care nurses (*N* = 229).

Attitude item	Response category	*n* (%)
*Advantages of integrating genetics into practice*		
Better treatment decisions	No advantage	45 (19.7)
	Advantage	184 (80.3)

Improved services to patients	No advantage	81 (35.4)
	Advantage	148 (64.6)

Better adherence to clinical recommendations among patients	No advantage	68 (29.7)
	Advantage	161 (70.3)

*Perceived disadvantages of integrating genetics into practice*		
Would take too much time	No disadvantage	207 (90.4)
	Disadvantage	22 (9.6)

Not reimbursable/too costly	No disadvantage	156 (68.1)
	Disadvantage	73 (31.9)

Need to “re‐tool” professionally	No disadvantage	188 (82.1)
	Disadvantage	41 (17.9)

Increase patient anxiety about risk	No disadvantage	181 (79.0)
	Disadvantage	48 (21.0)

Would increase insurance discrimination	No disadvantage	102 (44.5)
	Disadvantage	127 (55.5)

*Note:* Data are presented as frequency and percentage, *n* (%).

The highest reported disadvantage attitude toward integrating genetics of common diseases into practice is that it would take too much time, with a percentage of 90.4% (*n* = 207/229). The second highest reported disadvantage of integrating genetics of common diseases into practice attitude is that integrating genetics and genomics assessment may increase insurance discrimination with a percentage of 55.5% (*n* = 127/229) (Table 3).

### 4.4. Confidence of Genetics

Nurses demonstrated high levels of confidence overall, with the highest scores reported for accessing reliable and current genetic information and providing patients with information about genetic testing risks (both 86%, *n* = 197). Similarly, more than 80% of participants expressed confidence in multiple domains of genomic practice (Table 4). In contrast, lower confidence was observed in tasks requiring deeper clinical application, particularly in discussing how family history affects recommended screening intervals (65.5%, *n* = 165). Notably, this item also had the highest proportion of nurses reporting no confidence (34.5%, *n* = 79), indicating a specific gap in translating genomic knowledge into screening‐related decision‐making.

**TABLE 4:** Confidence on genetics and genomics in nursing practice of the study sample (*N* = 229).

No.	Confidence item	Not at all confident, *n* (%)	Confident, *n* (%)
1	Decide what family history information is needed to determine a patient’s genetic susceptibility to common diseases.	62 (27.1)	167 (72.9)
2	Discuss how family history affects recommended screening intervals.	79 (34.5)	150 (65.5)
3	Decide which patients would benefit from referral for genetic counseling and possible testing for susceptibility to common diseases.	42 (18.3)	187 (81.7)
4	Access reliable and current information about genetics and common diseases.	32 (14.0)	197 (86.0)
5	Give patients information about the risks of genetic testing for common diseases.	32 (14.0)	197 (86.0)
6	Give patients information about the benefits of genetic testing for common diseases.	37 (16.2)	192 (83.8)
7	Give patients information about the limitations of genetic testing for common diseases.	55 (24.0)	174 (76.0)
8	Facilitate referrals for genetic services for common diseases.	43 (18.8)	186 (81.2)

### 4.5. Decision/Adoption of Genetics

The decision/adoption domain is the utilization of family history information in the past 3 months and documentation of practice integration. Unfortunately, 82.1% (*n* = 188/229) reported that they rarely or never collected a complete family history in the past 3 months, and 17.9% (*n* = 41/229) reported occasionally collecting a full family history in the past 3 months. Furthermore, 99.6% (*n* = 228/229) of the participants failed to discuss genetics in the past 3 months. Most of the sample (87.3%, *n* = 200) picked the choice *never* regarding used family history information when facilitating clinical decisions or recommendations for the patients and facilitating referrals to genetic services (Table 5).

**TABLE 5:** The decision/adoption of genetics and genomics among critical care nurses (*N* = 229).

Patients that you have seen in the past 3 months	*n* (%)
*How often have you used family history information when facilitating clinical decisions or recommendations for your patients?*	
Never	190 (83)
Rarely	39 (17)
Occasionally	(0)
Frequently	(0)

*How often have you facilitated referrals to genetic services?*	
Never	200 (87.3)
Rarely	26 (11.4)
Occasionally	1 (0.4)
Frequently	2 (0.9)

These findings indicate a substantial gap between nurses’ genomic knowledge and the practical application of genomics in clinical care. Limited collection of comprehensive family history may reduce opportunities for early identification of hereditary conditions, timely genetic referrals, risk stratification, and personalized interventions, particularly in ICU settings where rapid clinical decision‐making is critical. Additionally, although female nurses demonstrated significantly higher genomic knowledge scores than male nurses, the underlying reasons for this difference remain unclear and may relate to variations in educational engagement, learning opportunities, or professional interests in genetics and genomics.

### 4.6. Social Subscale

The majority responded *yes* on the items: *Do you intend to learn more about genetics?* (89.5%, *n* = 205) and “Would you be able to attend a course during work hours?” (81.2%, *n* = 186). However, only 36.7% (*n* = 84) agreed that senior staff members see genetics as an important part of their role, and 31.9% (*n* = 73) agreed that senior staff see genetics as important to their own role. Notably, 37.6% (*n* = 86) responded *do not know* regarding whether senior staff view genetics as part of their role. While all nurses expressed a need to learn more about genetics, perceived supervisory support for genomic integration was limited (Table 6).

**TABLE 6:** Social domain of genetics and genomics among critical care nurses (*n* = 229).

Learning more about genetics and its application to your professional practice:	*N* (%)
*Do you intend to learn more about genetics?*	
Yes	205 (89.5)
No	24 (10.5)
Do not know	0

*Would you be able to attend a course during work hours?*	
Yes	186 (81.2)
No	41 (17.9)
Do not know	2 (0.9)

*Would you attend a course on your own time?*	
Yes	146 (63.8)
No	62 (27.1)
Do not know	21 (9.2)

*Do you think your senior staff members see genetics as an important part of your role?*	
Yes	84 (36.7)
No	98 (42.8)
Do not know	47 (20.5)

*Do you think your senior staff members see genetics as an important part of their role?*	
Yes	73 (31.9)
No	70 (30.6)
Do not know	86 (37.6)

### 4.7. Variables of the Survey Based on Nurses’ Demographics

The section now follows a clear structure: gender differences (significant), educational level (not statistically significant), hospital type (not statistically significant), correlations with continuous variables (age, experience, time spent in patient care), and the significant correlation between knowledge and confidence.

A statistically significant difference in knowledge scores was seen between male (*M* = 7.74, SD = 1.99) and female nurses (*M* = 8.31, SD = 1.95; *t* (227) = −2.199, *p* = 0.029), with female nurses exhibiting superior knowledge of genetics and genomics. Despite being statistically significant, the disparity in mean scores is minor, indicating that although gender may correlate with knowledge levels, its practical value is likely limited. This discovery may indicate variations in participation in continuing education or exposure to genomics‐related material, necessitating more investigation.

No statistically significant differences in knowledge scores were found based on educational level (BSN vs. MSN; *p* = 0.723) or hospital type (governmental, private, educational; *p* = 0.10), suggesting that gaps in genomic knowledge may be prevalent across various educational backgrounds and institutional contexts, rather than limited to particular subgroups.

Correlation analysis revealed no significant associations between knowledge scores and age, years of experience, or workload, indicating that clinical exposure alone may be inadequate for enhancing genetic knowledge, necessitating tailored educational interventions. A statistically significant positive connection exists between confidence and genetic knowledge (*r* = 0.197, *p* = 0.003), suggesting that nurses with enhanced understanding are likely to express more confidence. Nevertheless, the association remained tenuous, indicating that confidence may not entirely represent actual knowledge, and both areas should be incorporated into training programs.

Significantly, despite reasonable levels of understanding, this did not manifest in practical application. No notable difference was observed in knowledge ratings pertaining to decision‐making behaviors, such as family history evaluation and referral to genetic services. The disparity between knowledge and practice holds clinical significance. Insufficient gathering of family history may result in the failure to identify patients at genetic risk, postponement of diagnosis, and inadequate preventive therapy. Likewise, inadequate referral rates to genetic services may restrict access to expert evaluation and counseling, eventually impacting patient outcomes.

## 5. Discussion

To the best of our knowledge, this study contributes to the limited literature examining nurses’ knowledge, attitudes, and practices regarding genetics and genomics in ICU settings [14, 15]. To provide regional context, our findings can be compared to a previous study conducted in Northern Jordan. Almomani et al. [15] assessed genetic knowledge among the general public and various healthcare professionals, including nurses, physicians, and pharmacists. While their study was not ICU‐specific, they similarly found significant knowledge gaps among healthcare professionals, with over half reporting that their knowledge came from medical courses rather than formal, dedicated genetics education. This comparison underscores that the need for enhanced genetics education is a national issue across healthcare disciplines and settings, including the ICU.

Abad and Sur’s [16] research provides insights into how nursing is integrating genetics and genomics in a developing country’s healthcare system. Although not ICU‐specific, the study underscores the global relevance and challenges of integrating genetics into nursing, facilitating comparative analysis. Research data justify generalizing gaps to diverse clinical environments; the ICU is presented as one of many settings where underprepared nurses routinely care for genomics‐relevant patients [6, 16, 17]. Yet, they highlight the need to address genetic knowledge gaps across diverse healthcare settings, including ICUs [18].

The findings indicate that ICU nurses demonstrated moderate genomic knowledge (mean score = 66.9%), alongside a generally positive attitude toward genomics (70.7%), but limited integration into clinical practice, as evidenced by the high proportion of nurses who reported never discussing genetics with patients (99.6%). Additionally, workload was identified as a major perceived barrier (90.4%). Despite these patterns, the relationship between knowledge and practice adoption was not statistically significant (*p* = 0.13), suggesting that knowledge alone may be insufficient to drive behavioral change in clinical settings. This finding aligns with prior literature indicating that favorable attitudes toward genomics do not necessarily lead to practice adoption when nurses face barriers such as limited training, lack of confidence, unclear role expectations, and competing workload demands [12]. Critical care nurses showed adequate knowledge regarding collecting family history and specific knowledge on the genomics of common diseases such as the cardiovascular system, diabetes mellitus, and cancer (8.03/12). In comparison, a 2014 study by Calzone et al., which used the same GGNPS survey with a large, national sample of nurses in the United States, also found knowledge gaps in family history collection. However, the U.S. sample demonstrated a slightly higher mean knowledge score (75%) compared to our sample’s 66.9%, potentially reflecting differences in the integration of genetics into nursing curricula between the two countries [18].

The findings consistently reveal similar strengths and weaknesses in genetic knowledge among Jordanian ICU nurses, aligning with both national and international trends [1, 15]. This highlights the need for changes in program accreditation, competencies, and licensure to enhance nurses’ knowledge in genetics and genomics, enabling them to better support patients and families [8]. These findings have important implications for nursing education, clinical practice, and healthcare policy. Integrating genomics competencies into undergraduate and postgraduate nursing curricula is essential to prepare nurses for precision‐based healthcare delivery. At the institutional level, healthcare organizations should establish structured genomic competency frameworks, continuing professional development programs, and clinical support systems that facilitate the integration of genomics into routine nursing practice, particularly in high‐acuity settings such as ICUs [6]. According to extensive surveys, many American nurses still have a limited ability to use genomes in clinical practice, even 20 years after the introduction of genomics competence. The majority indicate lack of access to postlicensure genomic training, limited exposure to genetics during their nursing school, and poor genomic expertise [19]. Similar deficiencies have also been documented in China, Spain, and other foreign settings, where nursing curricula’s genomics material is still dispersed, scant, and inconsistently evaluated [20]. Jordan is familiar with the absence of formal genetics instruction. A prior investigation by Gharaibeh et al. [7] concentrating on Jordanian nurses and midwives also indicated a considerable deficiency in formal genetics education and postgraduate training. Our research verifies that more than 10 years later, this educational shortfall endures, especially among ICU nurses, and continues to affect their proficiency.

While the nurses in our study saw the value in genomic education, they also recognized significant barriers. This is consistent with the findings of Calzone et al., who also reported that nurses internationally identified time constraints, costs, and concerns about increasing patient anxiety as major disadvantages to integrating genomics into practice [16, 21].

The ICU nurses in this study exhibited a predominantly favorable disposition toward the incorporation of genomics into their practice, with 70.7% believing it imperative to further their education. This degree of receptivity parallels the results of a U.S. study by Calzone et al., in which a majority of nurses recognized the significance of genomics, indicating that favorable views may serve as a prevalent prelude to the incorporation of genomics into nursing practice, irrespective of the context.

This suggests that adequate knowledge can influence attitudes, which in turn impacts nursing practice [22]. A gap exists in current training programs regarding the integration of genetic education and family history into everyday nursing tasks. To address this, qualitative research is necessary to explore nurses’ experiences, and the results should guide policy changes [3]. This research is positioned at the intersection of nursing practice, education, genetics, and genomics. Focusing on these key areas will enhance the impact and visibility of these findings within both academic and professional communities.

Despite demonstrating limited knowledge of common disease genetics, nurses in this study reported high confidence in accessing genetic information and explaining genetic testing risks to patients, revealing a potential discrepancy between perceived confidence and actual knowledge [1]. This discrepancy may be explained by several organizational and educational barriers, including heavy workload in ICU settings, insufficient hands‐on genomic training, unclear referral pathways for genetic services, limited interdisciplinary collaboration, and inadequate leadership support for genomic integration. Although nurses expressed confidence in accessing and communicating genetic information, this confidence may reflect theoretical familiarity rather than practical competency in applying genomics during patient assessment and care planning. In the United States, a multiethnic sample of registered nurses’ genomic competencies was evaluated. They revealed that most of the nurses in their sample lacked confidence in integrating and applying genomics, which contrasts with the higher confidence levels reported by our ICU nurses in Amman [23].

Current evidence suggests that nurses with higher genetic knowledge have better attitudes, confidence, and preparedness to use genomics in clinical practice. However, practice integration is weak, and the data do not clearly link knowledge to attitudes, practice change, or policy formation. Most research is cross‐sectional or short‐term pre‐ and posteducational interventions with low sustained practice outcomes or policy‐level impact. Thus, genomic knowledge, attitudes, confidence, and selected indicators of practice are positively associated, but their effects on long‐term practice integration and nursing policy need further study [12].

In comparison, Jordanian nurses demonstrated higher confidence in integrating genetics and genomics into their practice than their international counterparts. This confidence could be further enhanced by fostering critical thinking and decision‐making skills related to genetic challenges [24]. Despite these advancements, there remain notable gaps in the education and confidence levels of nurses regarding genomics practice. Studies indicate a discrepancy between the perceived importance of genomics and the confidence to apply it in clinical practice [18]. Addressing this gap through robust educational initiatives is critical.

### 5.1. Genetics and Genomics Decision‐Making

The decision‐making domain focuses on nurses’ use of family history and genetic integration in practice, including assessing family histories, identifying genetic disorder signs, discussing inheritance risks, and offering referrals to genetic services [25]. ICU nurses demonstrated poor practices in referrals and family history collection, despite recognizing its importance. This aligns with findings in Italian nurses, where a survey of 385 nurses conducted via Survey Monkey found that 76.1% were female, 33.5% held bachelor’s degrees, and 74.8% primarily focused on patient care. The study indicated that most Italian nurses viewed genetics as minimally relevant to their daily work and lacked confidence in utilizing genetic information [26]. Both studies demonstrate a disconnect between recognizing the importance of family history and actually incorporating it into routine practice, suggesting this may be an international phenomenon requiring targeted educational interventions. The limited integration of genetics‐ and genomics‐related activities in the present study, including limited referral and family history collection, is consistent with findings reported among nurses in other healthcare settings, including Italian nurses. This comparison suggests that limited clinical adoption of genomics is not unique to Jordanian ICU nurses. However, the findings also indicate that education should extend beyond theoretical knowledge to include practical guidance on family history assessment, referral pathways, and nurses’ roles in genomic care. Accordingly, targeted and practice‐oriented education, supported by institutional guidance, may help improve the integration of genetics and genomics into ICU nursing practice [15, 27].

### 5.2. Social Aspects of Genetics and Genomics

Most nurses in this study expressed a desire to learn more about genetics and attend courses during or outside of work hours. However, they reported that senior nurses play a passive role in genetic assessment or teaching. This aligns with findings from other studies, where senior staff did not view genetics as part of their role [17]. Targeted educational interventions for practicing nurses may improve genomic knowledge and confidence, supporting the need for structured training to enhance ICU nurses’ preparedness to integrate genetics and genomics into clinical care [8]. This reflects a broader willingness among nurses to expand their knowledge and practice in genetics and genomics. Recognizing the value of genomic knowledge is essential for improving care for critically ill patients with genetic histories. Policymakers and managers must provide the necessary infrastructure to support genetic integration into clinical settings. Education is the most commonly used strategy. However, combining education with leadership support may create a collaboration effect [9, 15].

Several strategies may help address the identified genomic knowledge and practice gaps among ICU nurses. First, nursing curricula should incorporate applied genomics content, case‐based learning, and family history assessment competencies. Second, healthcare institutions should provide ongoing continuing education programs tailored to critical care settings. Third, peer‐supported learning and mentorship models may enhance nurses’ confidence and practical genomic application. Finally, leadership engagement and institutional support are necessary to foster a clinical culture that values genomics‐informed nursing practice. Recent evidence also supports the effectiveness of innovative digital educational strategies. For example, Ceylan et al. [25] demonstrated that WhatsApp‐based genomic training significantly improved nurses’ genetic knowledge and awareness levels, highlighting the potential value of flexible and technology‐supported learning approaches in genomic nursing education.

The findings also suggest that organizational and leadership support is critical for successful genomic integration into nursing practice. Although nurses demonstrated positive attitudes toward genomics and strong motivation for additional education, uncertainty regarding leadership expectations and institutional priorities may hinder implementation. Therefore, policymakers, nursing managers, and healthcare leaders should support the development of genomic infrastructure, continuing education opportunities, and clearer professional role expectations related to genomics within ICU settings. Combining educational initiatives with active leadership engagement may create a more supportive clinical culture that promotes genomics‐informed nursing practice. Such support may contribute to better preparation of nurses to collect family history, assess genetic risk, identify patients or families who may benefit from genetic referral or testing, and engage in genetics‐informed clinical decision‐making when appropriate.

### 5.3. Limitations

This study has several limitations that should be considered when interpreting the findings. First, the cross‐sectional design captures data at a single time point, limiting the ability to establish causal relationships or assess changes over time. Second, the convenience sampling strategy from three hospitals in Amman may limit the generalizability of findings to nurses in other regions of Jordan or other healthcare settings. The response rate of 49.7% (229/460) introduces potential nonresponse bias, as nurses with particular interests in genetics may have been more likely to participate. Additionally, the data collection period coincided with the COVID‐19 pandemic (September–December 2020), which may have influenced response rates and practice patterns due to increased workload and stress. Self‐report bias is another limitation, as participants may have overestimated their confidence or knowledge. The epidemiological situation related to the pandemic may have affected both the response rate and the representativeness of the sample. Finally, the study was conducted in ICU settings only, and findings may not be transferable to other nursing specialties where genetic applications differ.

## 6. Conclusion

The study highlights the necessity of strengthening genetic and genomic competencies among nurses through structured educational and institutional initiatives. Given the increasing prevalence of genetically influenced diseases and the growing role of precision medicine, targeted genomic education programs are essential for improving nurses’ knowledge, confidence, and clinical application of genomics. Peer‐supported training models, simulation‐based learning, and academic–clinical partnerships may enhance practical competency and facilitate integration of genomics into ICU nursing practice. Additionally, healthcare organizations should establish leadership‐supported genomic practice frameworks and continuing professional development opportunities to ensure sustainable implementation of genomics‐informed patient care.

## Author Contributions

Mahmoud Issam Salameh contributed to the analysis of the results, implementation, and writing the original draft of the manuscript. Amani Anwar Khalil contributed to the design and implementation of the research and supervised the project.

## Funding

No funding was received for this manuscript.

## Conflicts of Interest

The authors declare no conflicts of interest.

## Data Availability

The data supporting the findings of this study are available upon request from the corresponding author. The data are not publicly available due to privacy or ethical restrictions.
